# Supplementary material for: MDACl2-Modified SnO2 Film for Efficient Planar Perovskite Solar Cells
Source: Molecules. 2023 Mar 15;28(6):2668. doi: 10.3390/molecules28062668 (PMC10056177; doi:10.3390/molecules28062668)
Supplement: Supplementary file 1 [file molecules-28-02668-s001.zip › molecules-2276438-supplementary.pdf]

Supporting Information:

$MDACl_2$-modified SnO_2 film for efficient planar perovskite solar cells

Yaodong Xiao, Xiangqian Cui, Boyuan Xiang, Yanping Chen, Chaoyue Zhao, Lihong Wang, Chuqun Yang, Guangye Zhang, Chen Xie, Yulai Han, Mingxia Qiu *, Shunpu Li * and Peng You *

College of New Materials and New Energies, Shenzhen Technology University, Shenzhen, 518118, China.

* Correspondence: youpeng@sztu.edu.cn (P.Y.); lishunpu@sztu.edu.cn (S.L.); qiumingxia@sztu.edu.cn (M.Q.)

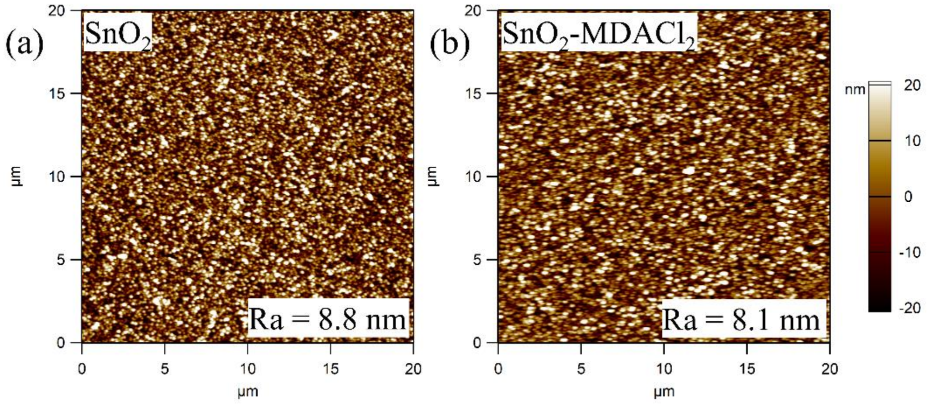

Figure S1. (**a,b**) AFM images of SnO_2 and SnO_2-$MDACl_2$ films grown on glass substrates.

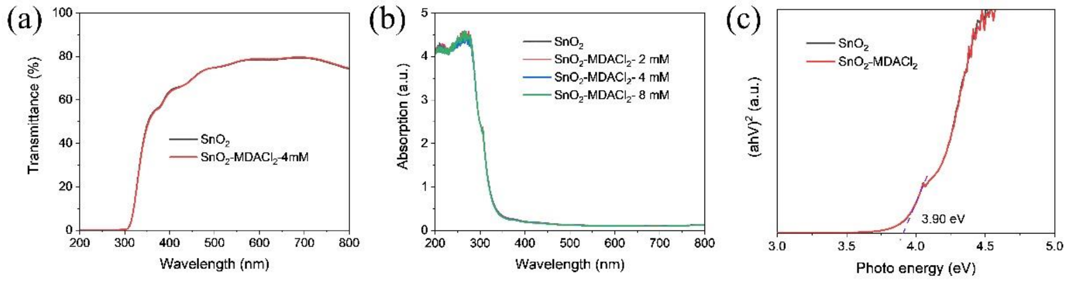

Figure S2. (**a**) Transmission spectra, (**b**) absorption spectra and (**c**) Tauc plots for the SnO_2 and SnO_2-$MDACl_2$ films.

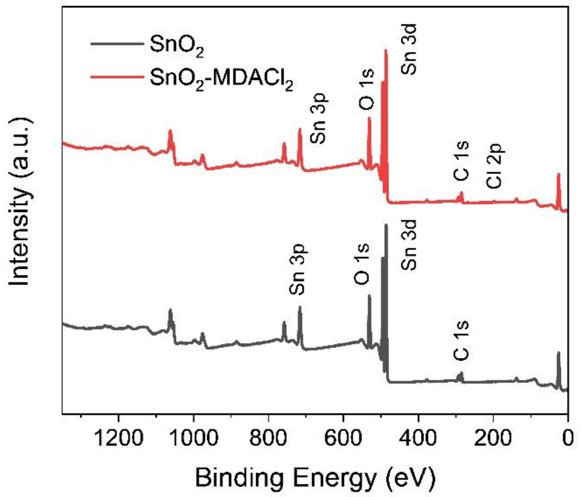

Figure S3. Full XPS spectra of the SnO_2, SnO_2-$MDACl_2$ films.

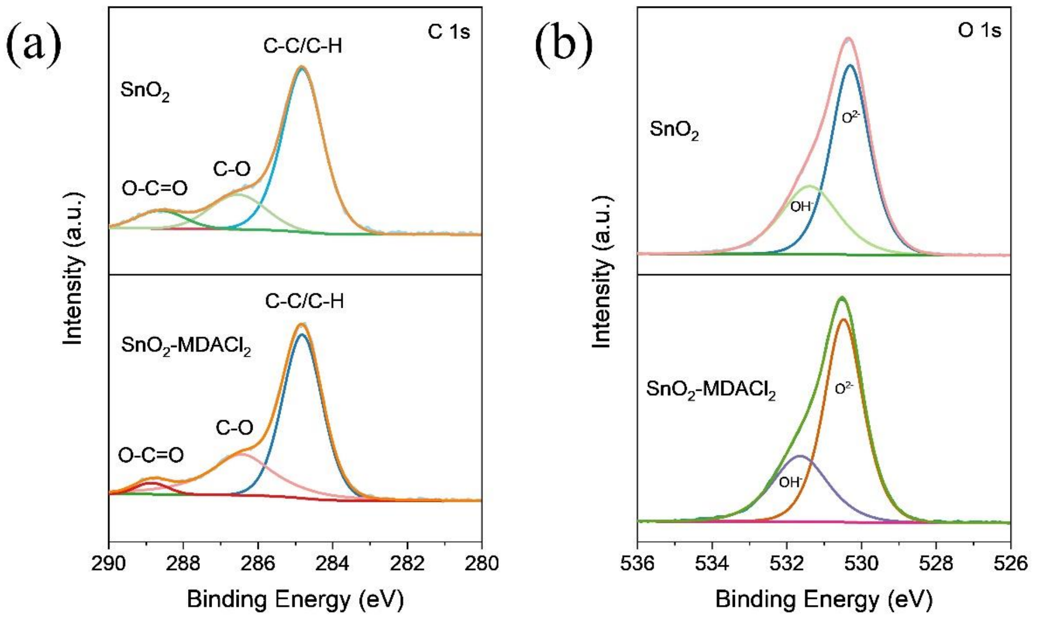

Figure S4. High-resolution (**a**) C 1s and (**b**) O 1s XPS spectra of the SnO_2 and SnO_2-$MDACl_2$ films.

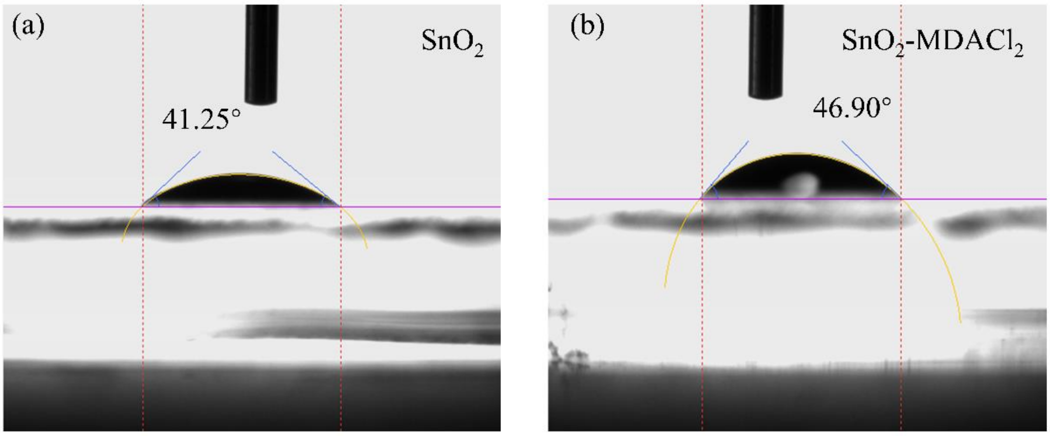

Figure S5. Contact angles of water droplets on the (**a**) SnO_2 and (**b**) SnO_2-$MDACl_2$ films.

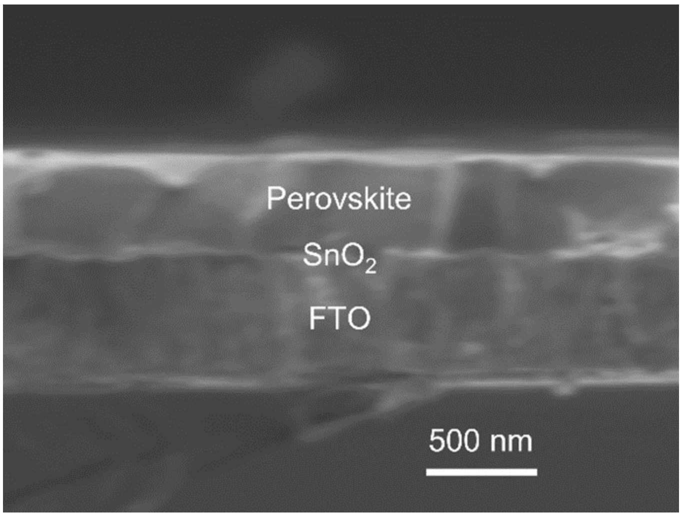

Figure S6. Cross-sectional SEM image of FTO/ SnO_2-$MDACl_2$/perovskite sample.

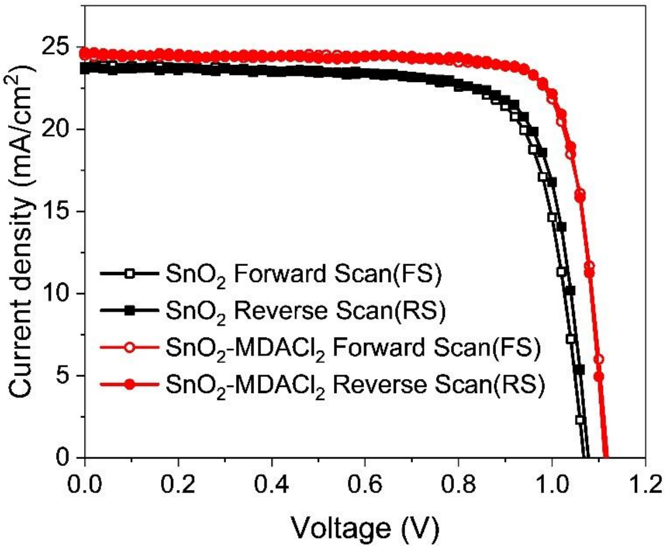

Figure S7. J-V curves of the PSCs based on SnO_2 and SnO_2-$MDACl_2$ ETLs under reverse scan and forward scans.

Table S1. Photovoltaic parameters of PSCs prepared by using $MDACl_2$-incorporated SnO_2 precursor solution and direct spin-coating $MDACl_2$ solution (4mM) on top of the SnO_2 film (statistical data of 20 devices for each conditions).

ETL	Voc (V)	Jsc (mA/cm^2)	FF (%)	PCE (%)
SnO_2	1.075±0.010	23.35±0.39	75.39±1.42	18.92±0.47
SnO_2+$MDACl_2$ (mixed precursor solution)	1.105±0.007	24.45±0.22	79.65±1.24	21.53±0.38
SnO_2/$MDACl_2$ (direct spin-coating)	1.098±0.009	24.09±0.14	77.10±1.23	20.41±0.44

Table S2. Band gaps (E_g), secondary−electron cutoff (E_{cutoff}), fermi level (E_F), valence band (E_{VB}) and conduction band (E_{CB}) for SnO_2 film and SnO_2-$MDACl_2$ films.

ETL	E_g (eV)	E_{cutoff} (eV)	E_F (eV)	E_{VB} (eV)	E_{CB} (eV)
SnO_2	3.90	15.72	5.50	8.29	4.39
SnO_2-$MDACl_2$	3.90	15.56	5.66	8.13	4.23

Table S3. Conductivities of FTO/SnO_2-$MDACl_2$/Ag with different doping concentrations.

ETL	SnO_2	SnO_2-$MDACl_2$ 2mM	SnO_2-$MDACl_2$ 4mM	SnO_2-$MDACl_2$ 8mM
Conductivity (mS/cm)	2.73×10^{-3}	4.26×10^{-3}	5.27×10^{-3}	4.13×10^{-3}

Table S4. Fitting parameters of the TRPL spectra for Glass/FTO/SnO_2/perovskite films. The average time constant τ_{ave} was calculated according to the equation: $\tau_{ave} = (A_1\tau_1^2+A_2\tau_2^2)/(A_1\tau_1 + A_2\tau_2)$.

ETL	A_1	τ_1 (ns)	A_2	τ_2 (ns)	τ_{ave} (ns)
SnO_2	0.625	**21.84**	0.457	**110.10**	**91.27**
SnO_2-$MDACl_2$	0.886	**11.67**	0.140	**91.23**	**55.64**

Table S5. Fitting values of R_s and R_{rec} of the corresponding devices in dark state

ETL	R_s (Ω)	R_{rec} (Ω)
SnO_2	37.16	217.3
SnO_2-$MDACl_2$	30.78	337.3
